# Implementation of the Operating Room Black Box Research Program at the Ottawa Hospital Through Patient, Clinical, and Organizational Engagement: Case Study

**DOI:** 10.2196/15443

**Published:** 2021-03-16

**Authors:** Sylvain Boet, Cole Etherington, Sandy Lam, Maxime Lê, Laurie Proulx, Meghan Britton, Julie Kenna, Antoine Przybylak-Brouillard, Jeremy Grimshaw, Teodor Grantcharov, Sukhbir Singh

**Affiliations:** 1 Department of Anesthesiology and Pain Medicine University of Ottawa Ottawa, ON Canada; 2 Clinical Epidemiology Program Ottawa Hospital Research Institute Ottawa, ON Canada; 3 Department of Innovation in Medical Education University of Ottawa Ottawa, ON Canada; 4 Francophone Affairs Faculty of Medicine University of Ottawa Ottawa, ON Canada; 5 Patient and Family Advisory Council The Ottawa Hospital Ottawa, ON Canada; 6 Main Operating Room The Ottawa Hospital Ottawa, ON Canada; 7 Department of General Surgery University of Toronto Toronto, ON Canada; 8 Li Ka Shing Knowledge Institute St. Michael's Hospital Toronto, ON Canada; 9 Department of Obstetrics, Gynecology, and Newborn Care University of Ottawa Ottawa, ON Canada

**Keywords:** patient safety, implementation science, quality improvement, health personnel, operating rooms

## Abstract

**Background:**

A large proportion of surgical patient harm is preventable; yet, our ability to systematically learn from these incidents and improve clinical practice remains limited. The Operating Room Black Box was developed to address the need for comprehensive assessments of clinical performance in the operating room. It captures synchronized audio, video, patient, and environmental clinical data in real time, which are subsequently analyzed by a combination of expert raters and software-based algorithms. Despite its significant potential to facilitate research and practice improvement, there are many potential implementation challenges at the institutional, clinician, and patient level. This paper summarizes our approach to implementation of the Operating Room Black Box at a large academic Canadian center.

**Objective:**

We aimed to contribute to the development of evidence-based best practices for implementing innovative technology in the operating room for direct observation of the clinical performance by using the case of the Operating Room Black Box. Specifically, we outline the systematic approach to the Operating Room Black Box implementation undertaken at our center.

**Methods:**

Our implementation approach included seeking support from hospital leadership; building frontline support and a team of champions among patients, nurses, anesthesiologists, and surgeons; accounting for stakeholder perceptions using theory-informed qualitative interviews; engaging patients; and documenting the implementation process, including barriers and facilitators, using the consolidated framework for implementation research.

**Results:**

During the 12-month implementation period, we conducted 23 stakeholder engagement activities with over 200 participants. We recruited 10 clinician champions representing nursing, anesthesia, and surgery. We formally interviewed 15 patients and 17 perioperative clinicians and identified key themes to include in an information campaign run as part of the implementation process. Two patient partners were engaged and advised on communications as well as grant and protocol development. Many anticipated and unanticipated challenges were encountered at all levels. Implementation was ultimately successful, with the Operating Room Black Box installed in August 2018, and data collection beginning shortly thereafter.

**Conclusions:**

This paper represents the first step toward evidence-guided implementation of technologies for direct observation of performance for research and quality improvement in surgery. With technology increasingly being used in health care settings, the health care community should aim to optimize implementation processes in the best interest of health care professionals and patients.

## Introduction

Over 50% of unintentional harm to hospitalized patients occurs in the operating room [1]. A large proportion of these incidents are preventable [1-3]. However, there has been no substantial reduction in patient safety events in recent years despite numerous advances in surgical and anesthetic practice and the proliferation of practice interventions [2,4]. Without tools that can be used to systematically observe health care provider performance in clinical practice and provide relevant and timely feedback, many interventions have been limited to the simulation environment [5-8]. Though valuable, simulation has many limitations and there is consensus that direct observation in the workplace is an extremely valuable method to assess clinical performance and to determine whether knowledge and skills transfer to practice [9].

The Operating Room Black Box was developed as a technological tool to address the need for a comprehensive understanding of clinical performance in the operating room. The Operating Room Black Box captures synchronized audio, video, patient, and environmental clinical data in real time, similar to black boxes in aviation [10]. Data captured by the Operating Room Black Box are subsequently analyzed by expert raters and software-based algorithms [10]. Accordingly, this makes it possible to study intraoperative performance without the need to be physically present in the operating room and to do so in a systematic way across a high number of surgical cases. This innovation maximizes opportunities to learn from and improve everyday practice. This leading-edge innovation also offers much needed transparency in a clinical environment that has traditionally been elusive [10,11].

When introducing tools to facilitate direct observation of clinical performance [12], particularly those that involve audio-video recording, there are many potential implementation challenges at the institutional, clinician, and patient level [12,13]. For example, there may be legal and ethical considerations, impact on workflow, and concerns about privacy, confidentiality, and evaluation [13]. Like any new health care technology, implementation may also have negative or unintended consequences if not undertaken systematically and sensitively [14]. Although much work has been done to establish best practices for implementing technology in health care, it has focused on the implementation of medical devices [15,16], electronic patient records [17-20], and simulation-based education technology [21,22]. Other studies have implemented technology for assessing performance, such as smartphone apps, and measured its impact without describing the implementation process [23]. There remains little implementation guidance regarding audio-video recording technologies designed to assess performance, especially in the operating room context.

With increasing calls for direct observation of clinical skills as part of postgraduate education and continuing professional development [24,25], it is essential to understand how tools designed for this purpose may be successfully implemented. In this paper, we outline the systematic approach to Operating Room Black Box implementation undertaken at our center. Our aim is to contribute to the development of evidence-based best practices for implementing innovative tools to directly assess performance in the operating room, using the case of the Operating Room Black Box.

## Methods

### Context

The Ottawa Hospital is one of Canada’s largest hospitals with a total of 1202 beds across 3 campuses. At the time of the study, there were 12,003 employees at the Ottawa Hospital along with 1481 physicians and midwives, 4440 nurses, 1194 residents and fellows, and 2201 researchers. Every year, approximately 35,000 surgical cases are performed at the Ottawa Hospital.

### Overview of Project Development and Implementation

The first phase of the project was a preimplementation period where the research team met with the hospital leadership and the developers of the Operating Room Black Box to determine feasibility and to establish an initial implementation plan. The research team also developed draft protocols for the first series of studies that would be conducted with Operating Room Black Box data and prepared funding applications to support implementation of the Operating Room Black Box and the planned research. The second phase of the project was the implementation period. The research team launched an information campaign to inform local stakeholders about the Operating Room Black Box and worked with the developers and local clinical managers, facility planning and support service staff, and information service technicians to install the technology.

We used a patient engagement approach to ensure that the project remained patient-centered at all times. Qualitative research was conducted throughout the project to inform and refine implementation strategies and to prospectively address challenges arising during the process.

### Preimplementation Period: Understanding Individual Stakeholder Perceptions With the Theoretical Domains Framework

To identify key issues to consider in Operating Room Black Box implementation, we conducted semistructured interviews with surgical patients, perioperative clinicians, and hospital administrators. The Theoretical Domains Framework (TDF) was used to inform interview guide development and data analysis. The TDF is comprised of 14 theoretical domains derived from behavior change theories that are relevant to behavior change (eg, knowledge, beliefs about capabilities, environment/resources) [26,27]. As one of the most commonly used frameworks in implementation research, the TDF is suitable for investigating potential barriers and facilitators to a particular behavior. In our case, we explored whether stakeholders (ie, patients, clinicians, administrators) would support research using the Operating Room Black Box.

A full description of the methodology has been published elsewhere [28]. Briefly, participants were recruited across each of the 3 campuses of our center, either in-person (patients) or via email (clinicians and administrators). Interviews were conducted by 2 trained interviewers who met regularly to discuss emerging themes as sample size was determined using the concept of data saturation. Saturation was defined as conducting a minimum of 8 interviewers per stakeholder group plus an additional 3 without the emergence of any new theme. In the case of hospital administrators, saturation was defined as conducting a minimum of 5 interviews plus an additional 3, given the small number of administrators at our center. Interviews were recorded, transcribed, deidentified, and imported into a qualitative analysis software (Nvivo 11, QSR International). Direct content analysis of the interviews was conducted in duplicate by 2 independent coders using a coding strategy based on the TDF. Data units (ie, several lines of text) were coded into themes within each of the TDF domains. Belief statements were generated based on these themes in order to represent common meaning across participant responses [27]. Domain relevance (ie, whether the domain/belief should be considered during implementation) was determined based on the perceived impact of the beliefs, the presence of conflicting beliefs within a specific domain, and the relative frequency of the beliefs across participant interviews [28]. Disagreements were resolved through consensus or consultation with a third researcher. Members of the research team with expertise in using the TDF along with practicing operating room clinicians reviewed the identified themes in order to ensure credibility of the data [29].

### Implementation Period: Identifying Barriers and Facilitators Across Individual and Organizational Levels With the Consolidated Framework for Implementation Research

The consolidated framework for implementation research (CFIR) [30] is a conceptual framework based on published implementation theories and reported studies. It includes 5 domains and 39 constructs that can be used “as a practical guide for systematically assessing potential barriers and facilitators in preparation for implementing an innovation” [30]. As a pragmatic model for implementation [31], the CFIR is specifically designed “to guide systematic research that supports rapid-cycle evaluation of the implementation of health care delivery interventions and produces actionable evaluation findings intended to improve implementation in a timely manner” [32].

As part of our systematic approach to implementation, we documented all aspects of the implementation process. All steps of the implementation were described and diagrams were generated to summarize each phase, including research ethics, legal and contract review, organizational parties involved, procurement, installation, data flow, consent, and information campaign strategies. This documentation provided an easy-to-follow reference point to share with stakeholders and to ensure all parties shared a common understanding of the implementation processes. We then used these documents to systematically identify themes relevant to implementation of the Operating Room Black Box across all stakeholder levels within the local context of our hospital. Barriers and facilitators were classified according to the CFIR. Direct content analysis of implementation documents was carried out by a member of the research team (SL) to identify and classify themes within the 5 CFIR domains: characteristics of the intervention, characteristics of the individuals involved, inner setting, outer setting, and process of implementation. Identified themes were then confirmed by 2 additional members of the research team (SB and CE).

Using the CFIR allowed us to prospectively develop strategies to overcome certain barriers and leverage facilitators at the systemic, organizational, and individual levels. In this way, we could address the practical needs of the stakeholders in charge of implementation of the Operating Room Black Box in our hospital as they arose.

### Patient Engagement Approach

To ensure that the Operating Room Black Box implementation was patient-centered, we worked closely with the Strategy for Patient-Oriented Research (SPOR) unit located at the Ottawa Methods Centre as well as the Patient and Family Advisory Council (PFAC) in the hospital. These existing groups provided our team with resources such as relevant training materials, which allowed us to conduct patient engagement using best practices. SPOR provided us with support on grant development, patient-advisor onboarding, and patient-engagement-evaluation surveys. We elected to recruit 2 patient advisors with lived surgical experience to the research team. PFAC aided in the recruitment of patient advisors and supported logistics for our patient-engagement activities and to overcome logistical barriers (eg, food vouchers and parking passes). While these practical details may not always be considered by research teams when engaging patients, they help to facilitate sustainable long-term collaboration with patients. We planned to engage patients in all key aspects of the implementation process, from developing communication materials to reviewing grant applications and study protocols. This would set the stage for continued patient involvement as our research using the Operating Room Black Box began.

## Results

### Preimplementation Period

**Figure 1:**
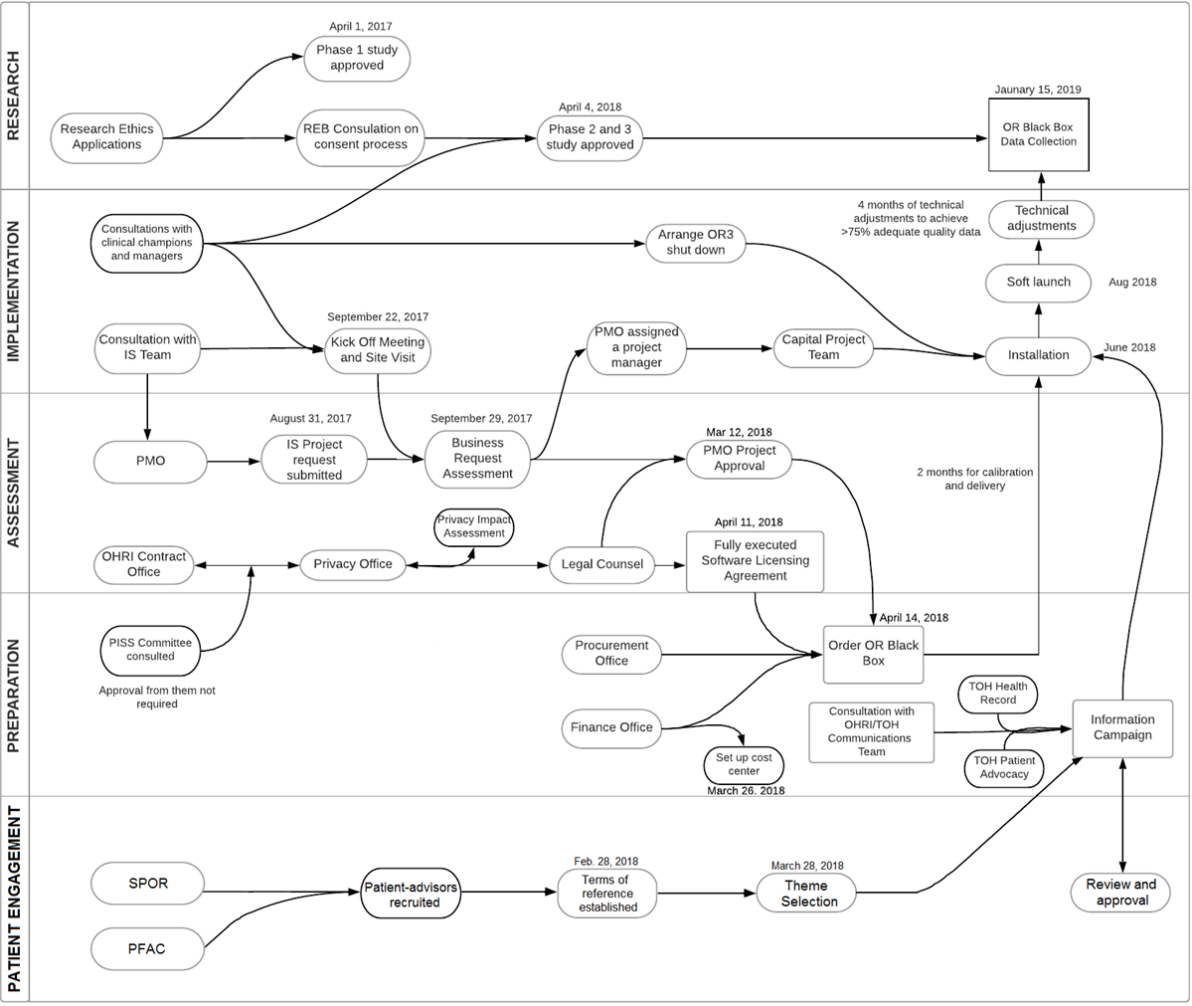
Implementation process map. IS: information services; OHRI: Ottawa Hospital Research Institute; OR: operating room; PFAC: Patient and Family Advisory Council; PISS: Privacy and Information Security Steering committee; PMO: project management office; REB: research ethics board; SPOR: strategy for patient-oriented research; TDF: theoretical domains framework; TOH: The Ottawa Hospital.

The overall implementation timeline and key activities are shown in Figure 1. The results of our experience of our implementation process are discussed below, including challenges and solutions to implementation of the technological tools studied, namely, the Operating Room Black Box.

### Seeking Support From Hospital Leadership

In December 2015, the principal investigator (SB) met with a key senior leadership team member to discuss the Operating Room Black Box concept. Next, in January 2016, the principal investigator presented the Operating Room Black Box concept to the corporate perioperative committee, which gathered key institutional clinical leaders in surgical specialties, nursing, and anesthesiology. The committee approved the idea of an Operating Room Black Box pilot and agreed to further consider this innovation for implementation in 1 operating room.

### Overview of Stakeholder Engagement Activities

We conducted consultation meetings and presentations for various stakeholder groups in addition to dissemination activities such as an e-newsletter. These activities took place on 23 formal occasions over a 12-month period, among many additional informal meetings. Various members of the core research team were involved in each activity, which was key to connecting with different audiences. Over 200 participants have been involved in these activities, indicating the wide reach of our implementation process at our hospital. Activities involved a wide range of stakeholders—from the Research Ethics Board (REB), who were essential to determining our approach to consent, to the Health Records and Patient Advocacy departments, who would respond to any patient’s request for their Operating Room Black Box recording. Multimedia Appendix 1 reports details on stakeholder engagement activities.

### Privacy, Confidentiality, and Ethics

**Figure 2:**
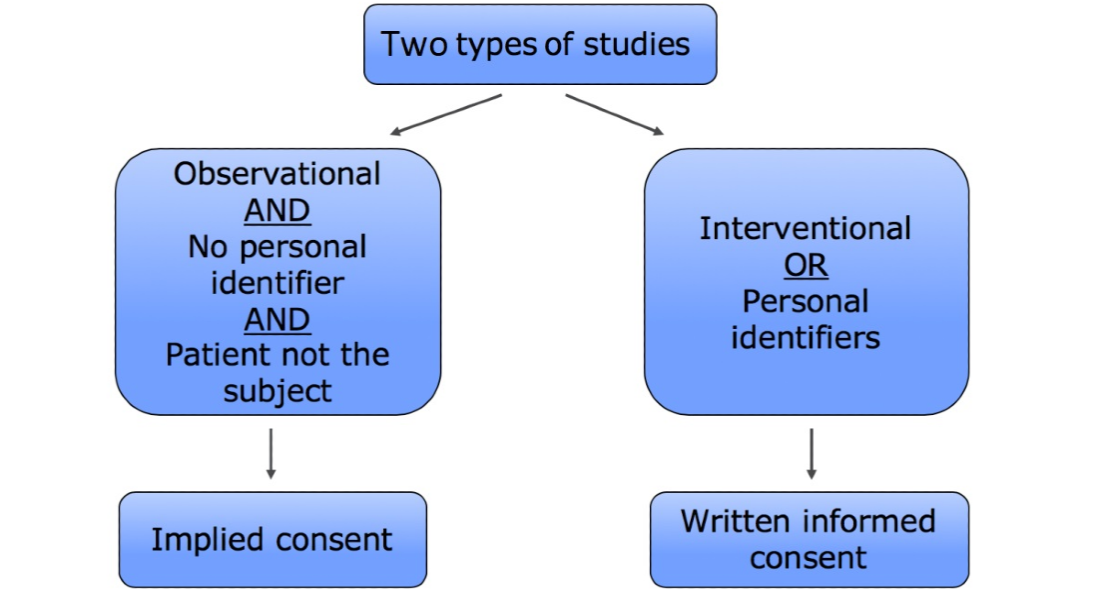
Operating Room Black Box Research Program approach to consent.

The Operating Room Black Box collects highly sensitive data both from the patient and the health care providers’ perspective. Privacy, confidentiality, and consent were top priorities. To develop an optimal ethical plan, we engaged clinicians, patients, and the Ottawa Health Science Network Research Ethics Board (OHSN-REB). The result was an approach to consent that would depend on the type of study involved (Figure 2). For observational studies that collected no personal identifiers and where the patient is not the subject of the research question, an implied consent approach is used. For interventional studies or studies that collect personal identifiers (eg, demographic information), a written informed consent is used.

The REB required that the implied consent approach was associated with an extensive hospital-wide information campaign. Information is first delivered to patients and clinicians prior to the surgery (through posters and pamphlets in the preoperative assessment unit, visited by patients in the weeks prior to their surgery, and institutional email). There is also a sign on the door of the room where the Operating Room Black Box is installed, and the confirmation of recording has become part of the preoperative team safety briefing. Patients and clinicians have 4 opportunities to opt out of being recorded before or within 48 hours of the surgery. It was also determined with the REB that critical situations would always be included as they represent important opportunities for learning and it would not be possible to determine who was in the room, given the high number of health care providers who would enter and leave the area, all of them wearing surgical masks. Of course, providers could still withdraw their recording up to 48 hours afterwards.

### Building Frontline Support and a Team of Champions

Through informal discussions, the principal investigator—a staff anesthesiologist and researcher —shared information about the Operating Room Black Box and the research vision with colleagues in anesthesiology, nursing, surgery and perioperative clinical managers and department heads. Motivated individuals from each provider group who were supportive of the idea were invited to become research collaborators and clinician champions. We recruited 10 clinician champions in total, representing nursing, anesthesia, and surgery. These champions facilitated implementation by communicating pertinent information about the project and the technology to their colleagues and informally building support within their professions. With the assistance of our clinician champions, we also developed an internal website. The website hosted a frequently asked questions page, research information page, newsfeed, and a video featuring champions discussing the purpose and importance of the Operating Room Black Box research program.

Using a participatory and collaborative approach, our collaborators and champions contributed to the development of grant applications and study protocols via integration of their professional experience and perspectives. This approach aimed to strengthen our relationship with the collaborators while encouraging them to take ownership of the project through research engagement. As clinician champions, these individuals continued to discuss the Operating Room Black Box concept with their colleagues and were key to relaying important information to frontline staff. Champions helped to answer questions on the frontline and generate support.

### Stakeholder Perceptions: TDF Interview Results

Our qualitative study of stakeholder perceptions regarding the Operating Room Black Box was approved by the OHSN-REB, Protocol #20170117-01H. This study was reported separately elsewhere [28]. In short, interviews were conducted with 15 patients across 3 hospital campuses (5 patients each), 17 perioperative clinicians, and 9 hospital administrators. Overall, patients and administrators had positive perceptions toward the Operating Room Black Box. Patients viewed the technology as a tool for their safety and as something to be expected at a teaching hospital that they trusted. Still, they expressed the importance of being provided with clear information on the Operating Room Black Box research prior to their surgery. Administrators indicated support for the Operating Room Black Box based on its perceived fit with the institutional mandate and the expectation that using the device for research would lead to improvements in patient safety culture, processes of care, and outcomes. They also emphasized the importance of appropriate implementation and ensuring the recordings were not used punitively. Compared to patients and administrators, health care providers reported more mixed perceptions toward the Operating Room Black Box. Feelings ranged from enthusiastic support for the potential of the Operating Room Black Box to feeling skeptical or threatened. Many questions were raised about its purpose, logistical implications, and potential negative impact on the dynamics in the operating room. A desire for more information was repeatedly expressed. Clinicians were most concerned about privacy/confidentiality and possible medicolegal repercussions. Still, many reported that the Operating Room Black Box fit with their role as health care providers, aligned with institutional values, and was necessary for progress and learning. Providers also expressed their willingness to participate in the research because of their trust in the principal investigator. Based on the results of this study, we created an internal website with a “Frequently Asked Questions” page to provide more information to stakeholders, addressing their unique concerns and drawing on the perceived benefits of the Operating Room Black Box that they highlighted during the interviews.

### Patient Engagement Results

Our collaboration with 2 patient advisors (ML and LP) began with the co-design of terms of reference, established between the patient advisors and the research team. The advisors were asked to assist in the development of the project and to collaborate with the research team in the development of a communications strategy. From the aforementioned interviews, the research team found 82 themes in the preliminary analysis. The researchers then presented patient advisors with a reduced number of 34 themes based on the frequency each was mentioned and perceived importance. From these, the patient advisors isolated the top 10 key themes that they determined important to convey to surgical patients and family/caregivers through communication materials that would be part of Operating Room Black Box implementation. The patient advisors collaborated in pairing patient messages with relatable graphic images and wording. This initial design session provided a framework for an informational patient poster (Multimedia Appendix 2) and pamphlet (Multimedia Appendix 3), which were then created through an iterative review process and interdisciplinary collaboration (eg, clinicians, researchers, hospital communications team, patients). Patient advisors ensured that the information and format would be relevant and easily understood by a lay audience, and their feedback influenced both the design of the materials and their placement in the care pathway (ie, made available to patients before the day of surgery). The materials created are currently available to patients in various locations at The Ottawa Hospital campuses. Of note, the design session followed an established design thinking process, which is a solution-focused methodology that employs divergent and convergent thinking of practical and creative solutions for the problems [33]. Together with the patient partners, we developed a study protocol and grant proposals for engaging patients in surgical safety research. The patient advisors provided a valuable perspective of how best to engage with patients prior to surgery and insight on communicating patient perspectives to hospital staff through newsletters and our internal website. Finally, the patient advisors engaged with the local media, the hospital newsletter, and the broader research community to share their experiences of being involved in Operating Room Black Box implementation and research projects.

### Multi-Level Barriers and Facilitators: CFIR Results

Below, we report the implementation barriers and facilitators identified within each of the 5 major domains of the CFIR:

1. Characteristics of the intervention: This domain is composed of core components (the essential and indispensable elements of the intervention) and adaptable periphery (adaptable elements, structures and systems related to the intervention and the targeted organization) [31]. The lack of published evidence on the use of this intervention and its potential cost may impede certain organizations from adopting the Operating Room Black Box. At the same time, however, the Operating Room Black Box is highly adaptable and testable on a small scale in the hospital, and there is no other alternative solution to compete against it. These conditions made it favorable to implement the intervention.
2. Outer and inner settings: The outer setting refers to the economic, political, and social context surrounding an organization, whereas the inner setting refers to the structural, political, and cultural context surrounding the implementation process. The Operating Room Black Box had a favorable outer setting in that the hospital acknowledged patient safety as a top priority and has a close network with other hospitals who have adapted the same technology. In addition, the concept of the Operating Room Black Box supports the CanMEDS Physician Competency Framework [34], which is a well-recognized framework in the Canadian physician population. With regard to the inner setting, there was a favorable culture to support research as well as strong leadership engagement. The team also conducted an information campaign to create an implementation climate that facilitated access to information and boosted the receptivity of the involved individuals to the Operating Room Black Box. Barriers in the inner setting included competing with other existing projects for budget and resources and the lack of a working model between the hospital and its research institute. As a result, significant time was invested by the Research Manager to liaise between the 2 institutions. The Research Manager subsequently worked with the Vice President of Innovation and Quality at the hospital to improve the processes in the future.
3. Characteristics of the individuals involved: This domain describes the perceived control, attitudes, norms, and intentions of individuals impacted by the intervention—in this case, surgical patients and staff. An interview study was conducted prior to Operating Room Black Box implementation to study the perceptions of these individuals. It was found that patients had positive beliefs toward the use of Operating Room Black Box to improve patient safety, and staff identified themselves with the hospital’s commitment to improve patient safety and care, both of which contributed to a receptive environment during implementation. The interviews also revealed some questions and misconceptions from staff about the intervention, which were used to develop key messages for the information campaign.
4. Process of implementation: This domain describes the active change process used to promote individual and organizational use of the intervention. It is composed of 4 essential activities: planning, engaging, executing, and evaluating. The Operating Room Black Box implementation revealed several major barriers related to planning, for instance, a lack of clear administrative process to follow for the implementation of innovation in the hospital, making it challenging to develop a comprehensive implementation plan. However, the team had built strong collaborations with the hospital’s capital project team and clinical departments, which greatly facilitated an effective engagement process and execution of the intervention.

The CFIR framework allowed us to assess current context through identifying major barriers and facilitators associated with implementing the Operating Room Black Box in a hospital setting. We were able to develop corresponding strategies to overcome certain barriers and play our strengths at the systemic, organizational, and individual levels. Details of barriers and facilitators using the CFIR tool are reported in Table 1.

**Table 1:** Barriers and facilitators to Operating Room Black Box implementation at the institutional level according to the consolidated framework for implementation research.

Domain, facilitators and barriers			Additional details
**Characteristics of the Operating Room Black Box intervention**			
	**Facilitators**		
		Adaptability: The platform is a highly adaptable. Its use can be tailored to local needs.	The research team secured grant funding for the purchase of the device. Long term maintenance is expected to be minimal.
		Trialability: The platform is implemented on a small scale (one operating room only) and is easily reversible.	Operation of the system is simple and completely unobtrusive.
		Relative advantage: There is no other existing intervention that could achieve the desired details and minimal intrusiveness offered by the platform.	N/A^a^
	**Barriers**		
		Evidence of strength and quality: New technology that lacks supporting evidence on its use to improving patient care.	Each institution has its own rules and structures related to information technology, which limited the team’s ability to draw on the experiences of other centers.
		Costs: There are costs associated with the purchase, installation, and maintenance of the equipment.	Before the approval of the project, there was no way for the research team to estimate costs associated with implementing the Operating Room Black Box at our institution.
**Outer setting**			
	**Facilitators**		
		Patient needs: Improving teamwork has been identified as a sustainable and practical way to promote patient safety.	There was a general positive environment in the outer setting that promotes the use of technology in improving patient care.
		Peer pressure: The platform has been successfully implemented in 4 other hospitals in Ontario.	Evidenced by successful implementation of the Operating Room Black Box nationally and internationally.
		Cosmopolitanism: Collaboration with experienced implementers to share best practices.	The lack of other alternatives to collect the same level of data in such an unobtrusive way also makes the Operating Room Black Box a favorable option.
		External policy and incentives: The concept of Operating Room Black Box supports the CanMEDS Physician Competency Framework.	N/A
**Inner setting**			
	**Facilitators**		
		Culture: Organizational commitment to support research to improve patient care.	Letters of support were received from the Chief Executive Officer and numerous department heads to secure grant funding to purchase the device.
		Readiness for implementation, leadership engagement: Overall strong support and commitment from leadership.	We established our network of support through early engagement with the senior leadership team (1 year prior to funding received).
		Access to knowledge and information: A comprehensive information campaign was in place to inform affected patients and clinicians of the intervention and how it would not affect their care.	Our information campaign included emails, posters, internal website, presentations at rounds, pamphlets, excerpts in internal newsletters, stakeholder meetings, etc.
		Implementation climate: The information campaign also aimed to promote positive momentum toward better practice and care through increased transparency and open discussions. Integration of Operating Room Black Box recording with existing work process. Patient advisors engaged early in the project design.	We have a structured opt-out process, which allows patients and clinicians to decline being recorded at 4 different time points. This strategy aims to increase transparency and to build a trusting relationship. This approach was developed in collaboration with clinician representatives and the Research Ethics Board.
	**Barriers**		
		Readiness for implementation, available resources: Concurrent budget cutting and other competing projects at the institutional level.	Lack of within-institution communication.
		Networks and communications: Lack of a working model between the hospital and research institute for implementation of new technology into clinical practice.	The research institute’s contract office faced many challenges related to the lack of an internal working model to collaborate with the hospital’s contract office and to determine who will be leading the negotiation of the project’s contract component. The Operating Room Black Box involves both research and clinical practice and therefore required approvals from both the Research Ethics Board and hospital administration. However, there was no standard procedure for the research team to follow.
**Characteristics and attitudes of clinicians, patients, and senior leadership**			
	**Facilitators**		
		Knowledge and beliefs about the intervention: Patients are open to the initiative.	Interviews with 15 surgical patients across the hospital’s 3 campuses confirmed support and appreciation for the Operating Room Black Box.
		Individual identification with organization: Shared staff commitment to improve patient safety and care.	Interviews with 17 perioperative clinicians and 9 hospital administrators identified a desire for progress and improving patient care (paper under final peer review). Patient advisors supported implementation.
	**Barriers**		
		Knowledge and beliefs about the intervention: Clinician skepticism regarding the value of new technology and perceived lack of trust in hospital management.	The interviews conducted also revealed that clinicians had many questions and misconceptions related to the use of the technology.
**Operating Room Black Box implementation process**			
	**Facilitators**		
		Engaging: Collaboration with the hospital’s capital project team on the installation.	A peer-to-peer approach in communicating Operating Room Black Box progress was particularly useful.
		Engaging: Use of an information campaign to ensure that all affected patients and clinicians are well informed.	Rather than sending out Operating Room Black Box communications through the research team, we collaborated with project champions and department leaders, who helped distribute Operating Room Black Box–related information.
		Executing: Use of soft launch to stress test the data collection protocol. Standardization of communication process for anticipated patient inquiries.	We believed that people were more responsive and felt more comfortable expressing their questions or concerns to their professional peers than to the research team directly. The research team ensured that any expressed concerns were addressed and that any required opt-out paper work was filled out, hence promoting a positive environment to discuss the Operating Room Black Box with professional peers, while minimizing the extra burden on our project champions.
		Planning and engaging: Kick-off meeting and regular newsletters.	N/A
		Early and ongoing engagement of patient advisors	N/A
		Communication strategy developed and accounted for various audiences.	We created a one-page process flow map and training materials to ensure that key actors and assessors were aware of the “big picture,” and the research team filled in the gaps when questions were raised.
	**Barriers**		
		Planning: Lack of knowledge of administrative process in the hospital. Unawareness of new committees and services that need to be informed of the Operating Room Black Box	N/A
		Executing: Participants are free to opt out from the program, making it impossible to predict participation rate. Implementation limited to some (not all) clinicians, creating multiple workflows for the same process. Hidden costs.	N/A

^a^N/A: not applicable.

### Implementation Period

The Operating Room Black Box implementation process was initially anticipated to be 6 months but it took 15 months due to unanticipated institutional barriers. A solution had to be found to each of these barriers, drawing upon implementation facilitators related to our planned research with the Operating Room Black Box and the technology tool installation. To allow for installation, the operating room in which the Operating Room Black Box was to be installed was closed for 7 days. The Operating Room Black Box was installed on June 29, 2018. On the door of the operating room, a sign informing all individuals that the device was present in that operating room was displayed (Multimedia Appendix 4). Following installation and testing, recording commenced in August 2018. Study data collection began in January 2019.

## Discussion

This paper summarizes our approach to implementation of the Operating Room Black Box at a large academic Canadian center. This approach may be useful to guide implementation of technological tools for direct observation of clinical performance at other centers. Specifically, our approach highlights the utility of engaging stakeholders early in the implementation process and identifying barriers and facilitators across individual and organizational levels. Key strategies for successful implementation of technological tools for observing clinical performance, based on our experience with the Operating Room Black Box, are summarized in Textbox 1.

**Key strategies for successful implementation of the technological tools for direct observation of clinical performance.**

- Engage stakeholders at the earliest phase and throughout the implementation process
- Assess all potential associated costs in advance of implementation
- Apply for installation funding
- Engage stakeholders at all levels (patients, frontline clinicians, hospital administrators, research, ethics, privacy, information technology, etc)
- Identify champions across professions
- Develop a user-centered communication and implementation plan (ie, tailored to patients and health care providers of local institution)
- Learn and map the local organization and roles and responsibilities across departments (eg, which offices need to be informed and involved)
- Include a “buffer” period in project timelines in case of unexpected delays
- Draw on implementation theory (eg, Theoretical Domains Framework, Consolidated Framework for Implementation Research)
- Develop collaborative research partnerships
- Document the implementation process
- Develop a patient engagement plan

When considering introducing technological tools for direct observation of performance in a clinical context, it is instrumental to engage with key stakeholders and funding agencies as early as possible in order to procure all necessary resources for implementation. Although we accounted for the cost of the tool itself, we did not anticipate several secondary costs associated with the project (eg, engineering time for modifications to the operating room, operating room closure during installation). Thus, it is critical to consider all potential costs prior to engaging in implementation of any technological tool in order to improve efficiency and reduce additional costs.

The partnerships we developed across stakeholder groups proved to be critical in obtaining all required approvals, finding solutions to barriers, and acquiring sufficient human and financial resources. Stakeholder engagement is a best practice that is applicable across technological tools. This is supported by the broader literature on implementing technology in health care [35] as well as guidelines in medical education for implementing methods of directly observing clinical practice [36]. In the case of the Operating Room Black Box, stakeholder engagement was central to the implementation success, particularly as challenges were encountered across multiple levels, from individual consent processes to regulatory systems.

During our implementation of the Operating Room Black Box, we simultaneously sought support from both senior leadership and frontline clinicians and made sure to listen to and incorporate feedback whenever possible. This facilitated a collaborative and solution-focused implementation approach. For example, our implied consent model emerged from consultation with the OHSN-REB, patients, clinical leaders, and frontline clinicians. Engaging frontline clinicians through a formal qualitative study and finding clinician champions also played a significant role during implementation, as concerns could be proactively addressed. Providing various opportunities for stakeholders to be heard and for stakeholders to receive information also helped to reduce any resistance that may have initially been present. Building a solid frontline support team may therefore be essential to creating positive momentum during the implementation phase of any technological tool. This type of approach could also facilitate knowledge translation and dissemination of results from the data gathered.

Beyond our collaboration with perioperative clinicians of all professions, administrators, and researchers, a second notable strength of our implementation approach was patient engagement. While patient engagement in other areas of health care has been well-documented [37], there is a clear knowledge gap regarding patient engagement in surgical patient safety research. This is problematic, in particular, given that patients are most vulnerable and unable to advocate for their own needs while under anesthesia. Our partnership with our patient advisors was essential for making our information campaign patient-centered and for aligning research priorities with those of patients. Significant efforts were made to plan for patient engagement activities, including detailing roles and responsibilities, timelines, level of effort, contributions, outreach, and evaluation. These evaluations allowed regular assessments to occur throughout the project and indicated both a positive experience by all team members and a meaningful impact on the project. Patient advisors also attended various local and international conferences, which helped to build momentum across institutions that are using or planning to use the Operating Room Black Box, fostering future collaborations to engage patients in surgical safety research on a larger scale.

Using technology for direct observation of clinical performance has many potential benefits for both patients and health care providers [11,38]. For example, data from the Operating Room Black Box system can be used to enhance training by learning from safety threats and resiliency supports. This, in turn, may improve processes of care and patient outcome. Of course, there are legal aspects of video and audio capture in health care, which warrant discussion. Although health care providers may have concerns about medicolegal risks, consultations with legal experts suggest recorded data would be protected under the *Healthcare Quality Improvement Act, 1986* (HCQIA) in the United States and the *Quality of Care Information Protection Act, 2016* (QCIPA) in Canada. The HCQIA protects medical professionals from prosecution for conduct that undergoes peer review, while QCIPA allows quality improvement matters, including critical incidents, to be openly discussed among health professionals. Essentially, each of these pieces of legislation aim to further improve the quality through open communication without fear of reprisal. As with any new form of data collection, time is needed to continue to study and address its implications. That being said, as long as technology such as the Operating Room Black Box continues to be implemented by and for frontline health care providers in partnership with patients, it is likely to result in innovative quality improvement that simultaneously protects operating room teams, hospitals, and patients—all working together toward the same common goal of quality of care.

We recognize that the approach described in this paper represents the implementation experience of a unique technological tool at 1 large academic Canadian center only and that there may be center-specific factors, which may be important to explore prior to implementation. Nonetheless, the overall general approach and documentation process that we report in this paper may be useful to other centers aiming to implement technological tools for direct observation of clinical performance in the operating room.

In conclusion, this paper represents the first step toward evidence-guided implementation of technologies for direct observation of performance for research and quality improvement in surgery. With technology increasingly being used in health care settings, the health care community should aim to optimize implementation processes in the best interest of health care professionals and patients.

## Appendix

Multimedia Appendix 1 Operating Room Black Box implementation: consultations and presentations.

Multimedia Appendix 2 Informational poster for patients.

Multimedia Appendix 3 Informational pamphlet for patients.

Multimedia Appendix 4 Poster on the operating room door.

## Abbreviations

CFIR: consolidated framework for implementation research
HCQIA: Healthcare Quality Improvement Act, 1986
OHSN-REB: Ottawa Health Science Network Research Ethics Board
PFAC: Patient and Family Advisory Council
QCPIA: Quality of Care Information Protection Act, 2016
REB: research ethics board
SPOR: strategy for patient-oriented research
TDF: theoretical domains framework
