# Supplementary material for: Implementation of the Operating Room Black Box Research Program at the Ottawa Hospital Through Patient, Clinical, and Organizational Engagement: Case Study
Source: J Med Internet Res. 2021 Mar 16;23(3):e15443. doi: 10.2196/15443 (PMC8074833; doi:10.2196/15443)
Supplement: Multimedia Appendix 1 [file jmir_v23i3e15443_app1.docx]

**Multimedia Appendix 1.** Operating Room Black Box implementation: consultations and presentations.

| **OR Black Box^®^ implementation: consultations and presentations** | | | |
| --- | --- | --- | --- |
| **Type of engagement** | **Presenter(s)** | **Date** | **# participants (estimated)** |
| Initial consultation with: |  |  |  |
| Research Ethics Board and contract | Research Associate (NE) and Research Manager (SL) | July 2017 | 5 |
| OR management | Research Associate (NE) and Research Manager (SL) | May 2017 | 4 |
| Audio-Video team | Research Manager (SL) | Jun 2017 | 2 |
| Privacy | Research Associate (NE) and Research Manager (SL) | Aug 2017 | 4 |
| Consultation with TOH communications | Research Associate (NE) and Research Manager (SL) | July 28, 2017 | 5 |
| Anesthesia working group first meeting | Principal Investigator (SB) | Sept 18, 2017 | 10 |
| Kick off meeting to set project timeline and confirm roles and commitment from key stakeholders | Principal Investigator (SB), Research Associate (NE) and Research Manager (SL) | Sept 22, 2017 | 35 |
| Consultation with The Ottawa Hospital patient advocacy | Research Associate (NE) and Research Manager (SL) | Oct 18, 2017 | 3 |
| E-newsletter to internal key stakeholders | Research Assistant (APB) | Oct 11, 2017 | 56 subscribers |
|  |  | Mar 20, 2018 |  |
|  |  | Jun, 2018 |  |
| Kick-off meeting with patient advisors to set terms of reference | Research team and patient advisors (ML, KP) | February, 2018 |  |
| Patient-led presentations to key research, clinical, and patient organizations (local and international) | Patient advisors (ML, LP) | October, 2018  November, 2018  January, 2019 |  |
| Grand round presentation to OR staff | Clinician Champion – Staff Surgeon (SS) | Mar 7, 2018 | Not available |
|  | Principal Investigator (SB) | Apr 25, 2018 | 21 |
|  | Clinician Champion – Staff Surgeon (SS) and Principal Investigator (SB) | May 30, 2018 | 15 |
| Insert in departmental newsletter | Principal Investigator (SB) | Dec 6, 2017 | Not available |
|  |  | Apr 20, 2017 | Not available |
| Weekly research news | Principal Investigator (SB) | Jun 4, 2017 | Not available |
| Nursing staff meeting | Research Assistant (APB) and Research Associate (NE) | Nov 11, 2017 | 10 |
| Presentation at corporate perioperative meeting | Principal Investigator (SB) | Jan 25, 2018 | 20 |
| Work in progress at research rounds | Research Associate (NE) and Research Manager (SL) | May 15, 2018 | 50 |
| Clinician information email | Research Manager (SL) | June 2018 | Not available |
| Trainee information email | Research Manager (SL) | June 2018 | Not available |
| Initial consultation with Health Records and Patient Advocacy | Research Manager (SL) | June 2018 | 2 |
