# Supplementary figures and images for: Implementation of the Operating Room Black Box Research Program at the Ottawa Hospital Through Patient, Clinical, and Organizational Engagement: Case Study
Source: J Med Internet Res. 2021 Mar 16;23(3):e15443. doi: 10.2196/15443 (PMC8074833; doi:10.2196/15443)

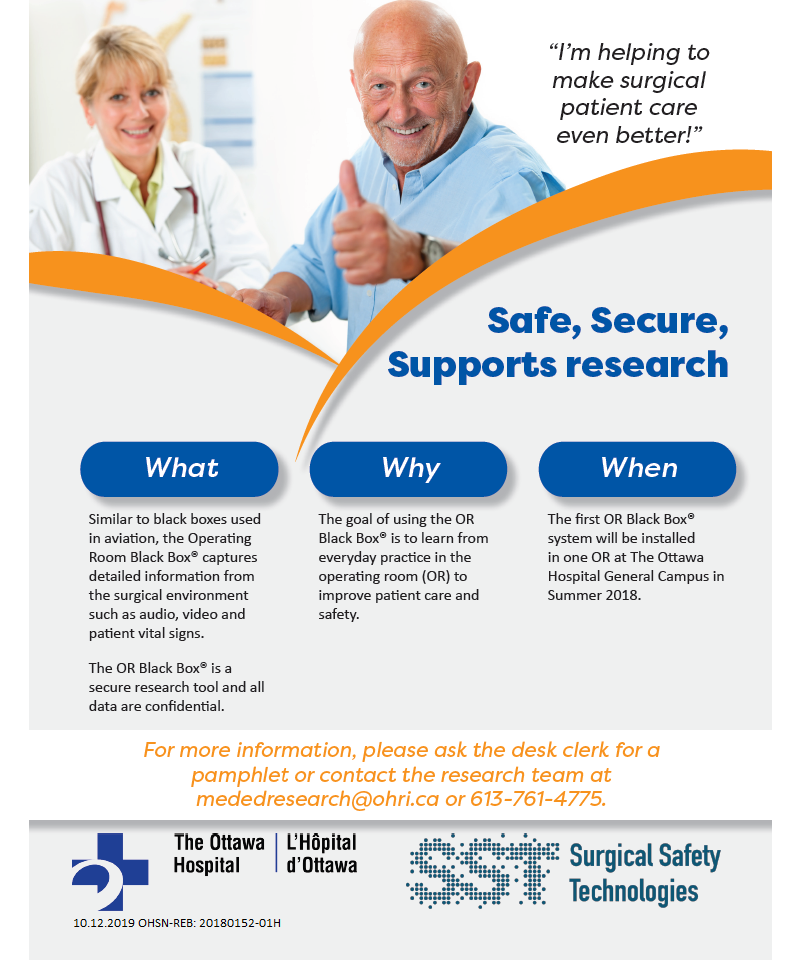

Supplement: Multimedia Appendix 2 [file jmir_v23i3e15443_app2.png]

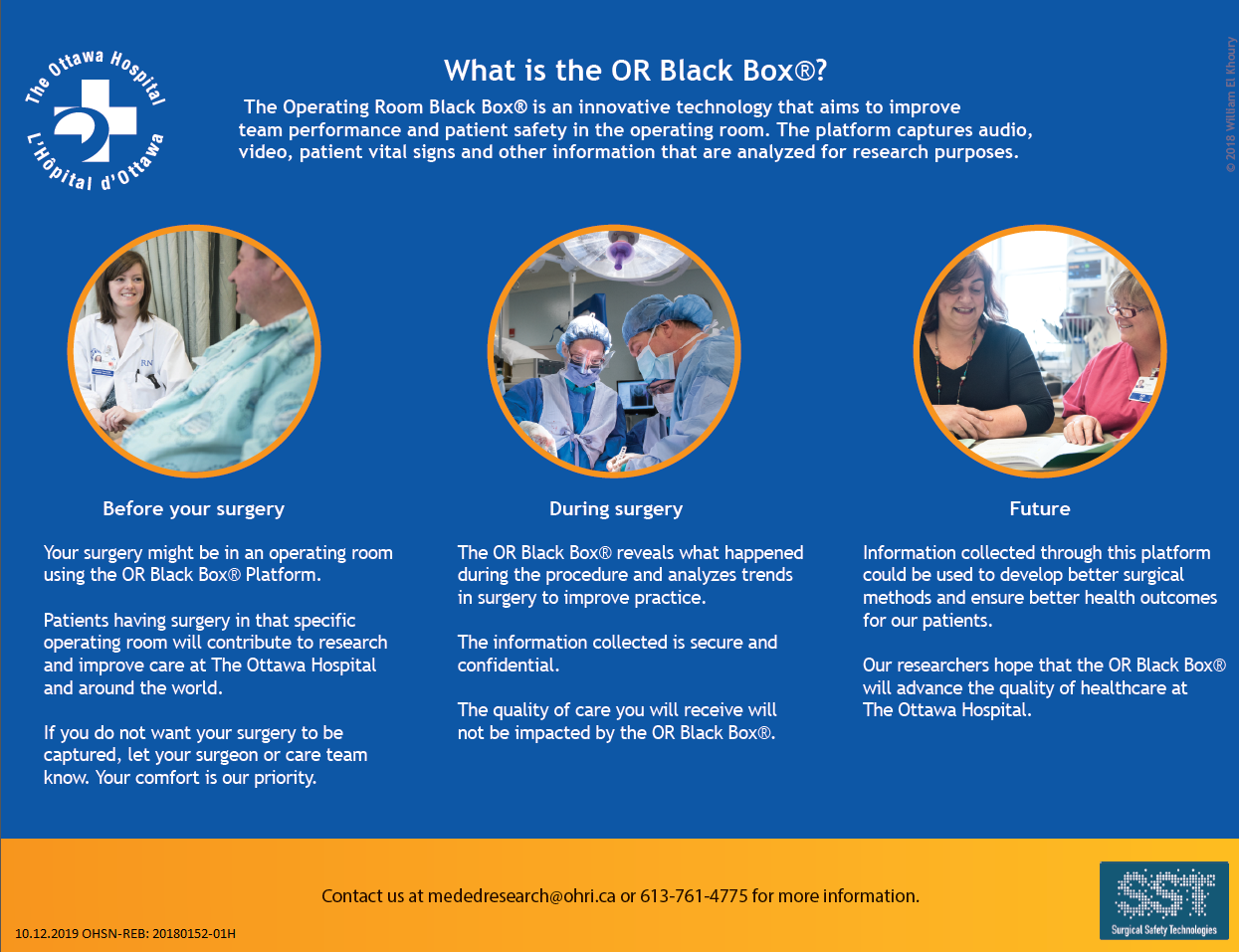

Supplement: Multimedia Appendix 3 [file jmir_v23i3e15443_app3.png]

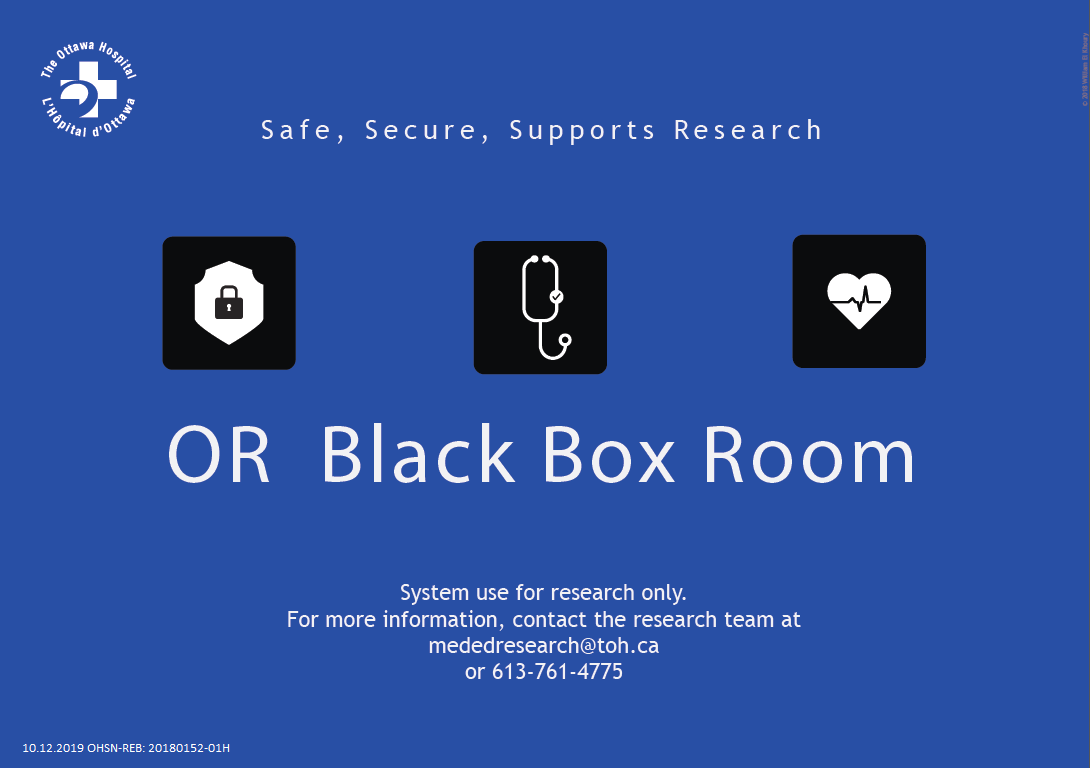

Supplement: Multimedia Appendix 4 [file jmir_v23i3e15443_app4.png]
